# Systematic overrepresentation of DNA termini and underrepresentation of subterminal regions among sequencing templates prepared from hydrodynamically sheared linear DNA molecules

**DOI:** 10.1186/1471-2164-11-87

**Published:** 2010-02-02

**Authors:** Sherri L Schwartz, Mark L Farman

**Affiliations:** 1 Department of Plant Pathology, University of Kentucky, 1405 Veterans Dr, Lexington, KY 40546, USA

## Abstract

**Background:**

Analysis of fungal genome sequence assemblies reveals that telomeres are poorly represented even though telomeric reads tend to be superabundant. We surmised that the problem might lie in the DNA shearing conditions used to create clone libraries for genome sequencing.

**Results:**

A shotgun strategy was used to sequence and assemble circular and linear cosmid DNAs sheared using conditions typical for a genome project. The DNA sheared in circular form assembled into a single sequence contig. However, the linearized cosmid produced an incomplete assembly because the two DNA termini, though greatly overrepresented in the clone library used for sequencing, were separated from neighboring sequences by gaps of ~1.4 and 1.8 kb. These gap sizes were reduced, but not eliminated, by shearing the linear cosmid into smaller fragments. Mapping of shearing breakpoints revealed a paucity of breaks in the subterminal regions of the linearized cosmid and also near chromosome ends of the fungus *Neurospora crassa*.

**Conclusion:**

Together, our data indicate that the ends of linear DNA molecules are recalcitrant to hydrodynamic shearing. We propose that this causes DNA termini to be overrepresented in the resulting fragment population but ultimately prevents their incorporation into sequence assemblies.

## Background

Searches for telomere-associated sequences in several fungal genome databases have been strikingly unfruitful [1]. Initially, we suspected that the chromosome ends may be recalcitrant to cloning or sequencing, or that they are not easily assembled, perhaps due to their repetitive nature. However, analysis of raw sequencing reads obtained from trace archives for several fungal genomes revealed that telomeric sequences occur in greater abundance than would be expected based on the average depth of sequence coverage. Once these sequences have been retrieved, they can usually be assembled into robust contigs with each contig representing a different chromosome end [1]. However, in most cases, the resulting telomeric contigs escape incorporation into the genome assembly because there are simply no sequence reads capable of connecting them.

This systematic underrepresentation of connecting reads pointed to a possible shortcoming in the "shotgun" sequencing strategy that is widely used to generate clones for genome sequencing. To date, most fungal genome sequences have been reconstructed from end-sequences of plasmids whose inserts were derived by hydrodynamic shearing of genomic DNA, selecting fragments in particular size-ranges and sub-cloning these fragments. Hydrodynamic shearing occurs when a DNA solution is injected at high speed through a narrow constriction. As the solution accelerates to maintain the flow rate, the DNA strands become stretched to a point where they break. This fragmentation continues through iterative shearing cycles until the drag forces on the resulting pieces become too small to overcome the tensile strength of the DNA strands. The end result is a population of fragments whose sizes are approximately normally-distributed, with a mean size that is determined by parameters such as orifice size, injection speed, as well as the viscosity and salt concentration of the DNA solution [2].

An important consideration when reconstructing a genome sequence is that shearing should be random, so that all DNA regions have an equal probability of being sufficiently close to a break point, that they gain representation among the shotgun sequence reads. However, studies indicate that hydrodynamic shearing causes DNA to break in a decidedly non-random fashion. Specifically, DNA strands tend to break near their midpoints with a standard deviation of ~12.5% of the molecule's length [3]. In addition, break points may be affected by sequence composition, with GC-rich regions appearing to be particularly susceptible to breakage [2].

**Figure 1:**
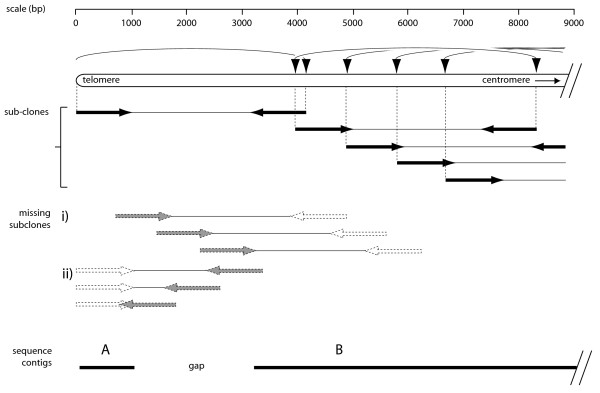
**Theoretical distribution of DNA breaks and resulting sequencing templates near a telomere**. The figure assumes that the shearing conditions were similar to those used in a typical genome sequencing project. Horizontal arrows represent sequencing reads derived from each end of a fragment whose termini correspond to the positions at which the original DNA strand broke under shearing stress. These positions are represented by vertical arrows. Breaks defining the two ends of a sequence template are connected with a curved line. The solid lines below the reads represent contigs resulting from assembly of the individual sequences. The breakpoint nearest to the telomere produces two DNA fragments, one telomeric and one subtelomeric. Both types of fragments potentially contain sequence information capable of linking the telomeric contig to the "internal" assembly. However, shearing the DNA to an average length of 4 kb should produce very few breaks in the region immediately adjacent to the telomere. Thus, the sequencing reads represented by dotted lines in (i) and (ii) are likely to be very rare. Furthermore, if a size-selection step is employed (as is typical for a genome project), the reads shown in (ii) will be further depleted. The scale at the top shows the distance from the telomere.

We surmised that the DNA shearing and size selection processes used in most genome sequencing projects might be problematical for the sequencing and assembly of telomeres. This is because, in a sequencing project where genomic DNA is typically sheared to a mean size of ~4 kb, midpoint breakage means that most breaks will have occurred at a distance of ~4 kb from the telomere, with very few breaks occurring in between. Consequently, unless the sheared DNA population has a wide size distribution, it will contain very few fragments capable of providing sequence that overlaps with the telomeric reads (Figure 1). Furthermore, the size selection process that is usually employed in genome projects should exclude the recovery of short terminal fragments, further depleting clones that could provide bridging sequences. Considering these issues, we hypothesize that hydrodynamic shearing is biased against breakage near DNA ends and this, in turn, prevents the assembly of terminal DNA regions in sequencing projects.

Computer modeling of DNA breaks near to DNA termini was performed in the late 1970s by Iyengar and Quave [4,5]. Their results suggested that breaks ought to be approximately evenly distributed throughout most of the DNA strand, except near the termini where breaks were predicted to be extremely rare [4]. However, until now, the computer models have not been experimentally tested. Therefore, to test the hypothesis that hydrodynamic shearing is biased against DNA termini, we performed shotgun sequencing of a single cosmid clone that had been linearized prior to shearing and mapped the positions of DNA breaks in the vicinity of the DNA termini. At the same time, we compared the quality of the assembly with one derived from the same cosmid in its circular form and with linear DNA that had been sheared to a smaller size range. Finally, raw sequence data from the *Neurospora crassa *genome project was analyzed to determine if bias against subterminal breakage could be responsible for the underrepresentation of telomeres in the shotgun assembly.

## Results

### Shotgun reads derived from linearized cosmid DNA resulted in an incomplete assembly with subterminal gaps

**Figure 2:**
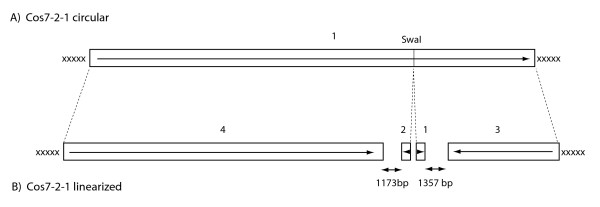
**Contigs resulting from the assembly of a cosmid clone sheared in circular or linear forms**. Contigs derived from the cosmid insert are drawn as white boxes. Sequences from the cosmid vector are shown in black. The lengths of the contigs and the sizes of gaps are shown. A) circular DNA sheared to an average size of ~3.8 kb; B) linear DNA sheared to an average size of ~3.8 kb.

To determine if shearing introduces a systematic bias against the assembly of subterminal DNA sequences, we utilized a shotgun strategy to sequence and assemble circular and linearized versions of a cosmid clone (Cos7-2-1). This clone contains sequences linked to the *BUF1 *gene of the fungus *Magnaporthe oryzae *(strain 2539) and was selected because it contains a single *Swa*I restriction site that could be used for linearization. The circular and linear DNA samples were separately sheared using the same speed setting, the sheared DNAs were size-fractioned by gel electrophoresis and fragments ranging from 3.8 to 4.2 kb were recovered from the gel and used to generate sequence-ready plasmid libraries. End-sequences were acquired, the vector backbone was masked out, and the remaining sequences were assembled. For the circular DNA, 1005 useful sequence reads were generated with average high quality (HQ) read length (Phred20) of 405 bp (this represented a coverage of ~10×). These reads assembled into a single 35,897 bp sequence contig which spanned the entire cosmid insert (Figure 2A). For the linearized cosmid DNA, 1,499 useful sequencing reads were generated, with an average high quality read length of 394 bp (>12× coverage). The *Swa*I cut site is located between positions 26114 and 26115 in the cosmid insert. Therefore, complete assembly ought to have produced two contigs of 26,114 and 9,783 representing the cleaved halves of the cosmid insert (the vector backbone having been masked out). However, the linearized cosmid assembled into four contigs whose lengths were 697, 667, 8426 and 24,276 bp (Figure 2B). The two smallest contigs (1 and 2) consisted of sequences derived from the two ends of the linearized DNA. Both contained an extraordinarily high number of reads - 103 and 108, respectively - and, in every case, the sequences read in a single direction - inward from the *Swa*I cut site. The fact that these terminal contigs lacked reads in the opposite direction confirmed the prediction made in Figure 1, example ii, that clones with short inserts would be efficiently excluded by size selection. The two small contigs were separated from the adjacent, and much longer, contigs by gaps of ~1.2 and 1.4 kb or, to use another relevant metric, approximately three read lengths.

### Distribution of breakpoints near to the ends of linearized cosmid DNA

**Figure 3:**
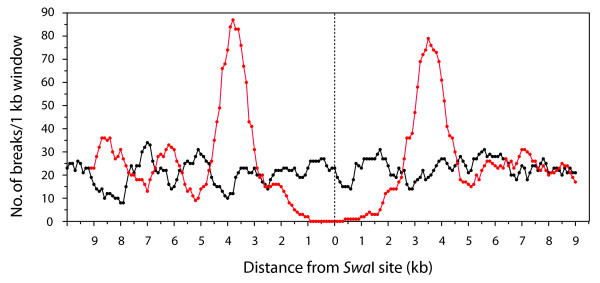
**Distribution of DNA breaks in the regions flanking the *Swa*I cut site**. The positions of breaks were determined by mapping the starts of sequence reads back to the cosmid assembly. Only reads that started with a visible pBC polylinker sequence were included. Shown are the results of a sliding window analysis of break distribution in the circular and linear DNA samples sheared to an average size of 3.8 kb. Window size was 1 kb and step size was 100 bp. The scale at the bottom represents the distance from the nearest window edge to the *Swa*I site.

The complete assembly of the circular cosmid showed that the sequences adjacent to the *Swa*I site are not recalcitrant to cloning. In addition, the average sizes of inserts in the "linear" and "circular" clone libraries were very similar (4045 bp, S. D. 487; and 3972 bp, S.D. 558), respectively. Therefore, the absence of reads that overlap with the sequences of the cosmid termini pointed to a deficiency of breaks near to the ends of the linearized DNA. To confirm this suspicion, we used a sliding window analysis to determine the distribution of breaks across a region spanning from 10 kb upstream to 10 kb downstream of the *Swa*I site. The "721 circular" assembly had 431 sequence reads that initiated within the 20 kb target region. Numerous breaks occurred in proximity to the undigested *Swa*I site (Figure 3A), indicating that the flanking regions aren't inherently resistant to cleavage. In addition the sliding window analysis revealed that the breaks were fairly evenly distributed throughout the whole region, with approximately 25 per kilobase.

The "721 linear" assembly contained 538 breaks that started within 10 kb of the cleaved *Swa*I site, with 307 mapping upstream and 231 mapping downstream. However, there were very few breaks within 2 kb of either terminus (only two on each side). The closest breaks to the *Swa*I site were 1.4 kb on one side and 1.8 kb on the other and, in both cases, the relevant sequences read away from the cut site, indicating that they were derived from subterminal fragments. This, again, was expected because the size selection process should have prevented recovery of terminal fragments shorter than 2 kb.

Interestingly, the sliding window analysis revealed that the density of breaks increased steadily after the far edge of the window reached the 2 kb mark and reached distinct peaks where the midpoint of the window was ~3.7 kb on either side of the *Swa*I site (Figure 3B) - a distance that corresponds approximately to the average size of fragments generated in the shearing process (see below). Beyond these maxima, both sides exhibited a slight dip in break density before leveling out at approximately 25 per kilobase.

To determine if shearing the linearized DNA down to a smaller size range would eliminate the subterminal gaps, we performed limited sequencing on DNA sheared using a speed setting of 6 which yielded DNA fragments with an average size of ~2 kb. A total of 378 useful reads were produced (average HQ read length = 434 bp) representing an ~4-fold sequence coverage. As expected based on the lower depth of sequencing, the "2 kb" fragments produced an incomplete assembly consisting of 8 contigs and, again, there were gaps between the contigs that contained the cosmid termini and the internal sequences. However, despite the lower level sequence coverage and the poorer assembly, the subterminal gaps were only 525 bp and 457 bp in length (results not shown).

### A standard shearing assembly rarely produces breaks within 1 kb of an existing DNA end

**Figure 4:**
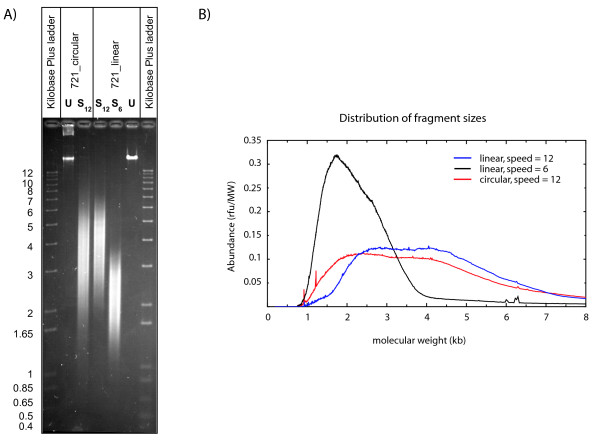
**Distribution of fragment sizes after shearing the circular and linear cosmid DNA**. Aliquots of the sheared DNA samples used to construct the sequencing libraries were run on a 0.7% agarose gel alongside a 1 kb plus size marker. The gel was stained with SybrGold and scanned in a FluorImager. Molecular abundance was measured using an arbitrary value which was calculated by dividing the fluorescence intensity by molecular length. A) shows the resulting digital image. B) shows a graph of the size distributions for the three sheared DNA samples.

Analysis of break distribution along the linearized DNA molecule revealed that the closest shear points were ~1.4 and 1.8 kb away from the termini. Clearly, breaks would need to occur considerably closer to the DNA end in order to link up the terminal sequence contigs. Indeed, considering that the longest reads achievable with current sequencing technologies are in the range of 1 kb, successful extension of a terminal sequence would necessitate that breaks occur ~800 to 900 bp from the molecule's end. To determine how frequently the shearing process produced breaks this close to DNA ends, we examined the size distribution of the fragments used to produce the Cos7-2-1 assemblies. An aliquot of each sheared DNA sample was fractionated by agarose gel electrophoresis, stained with SYBR Gold™ and the gel was then densitometrically scanned using a FluorImager. An image of the gel is shown in Figure 4A and fragment abundance estimated by densitometric scanning is graphed in Figure 4B. With speed setting 12 the most intense fluorescence was in the 4 kb size range. Correcting for the relationship between fragment size and fluorescence intensity revealed that the average size was ~3.8 kb, with fragments ranging from 2 to 4 kb being present in approximately equal abundance. Figure 4A shows that there were very few fragments less than 1.5 kb in length and, hence, very few breaks within 1.5 kb of the ends of DNA molecules. Calculation of the area under the curve in Figure 4B revealed that only 3.3% of breaks occurred within 1.5 kb of an end, 0.15% were within 1 kb and breaks less than ~800 bp away from an end were essentially undetectable above background. The circular DNA produced a very similar size distribution (Figure 4B), although the representation of fragments in the 1 to 1.8 kb size range was increased more than two-fold.

The faster shear speed setting produced much smaller fragments with the average length being ~1.9 kb. More importantly, the proportion of breaks within 1.5 kb of an end was 23%, or seven times greater than what was observed in the linear sample sheared at the slower speed setting (Figure 4B). This explains why the gap sizes were much smaller in the "2 kb" assembly. Even so, the "2 kb" fragment population exhibited a size cutoff at the same position as the "4 kb" populations, so that fragments less than ~0.8 kb were undetectable by fluorimetric scanning.

### Failure to shear DNA in the subterminal regions results in an overrepresentation of terminal fragments

The large number of sequence reads representing the linearized cosmid termini indicated that the ends of the DNA molecule were massively overrepresented in the clone library used for sequencing. We hypothesized that this is a direct result of failure to cleave in the subterminal regions. The rationale being that, if the ends of DNA molecules cannot be broken near to the termini, then the breaks that should have occurred in these regions will occur, instead, at the nearest cleavable location. To test this hypothesis, we counted the number of sequence reads in the regions spanning from each end of the linear cosmid to the positions of maximal breakage and compared these values to the expected number of reads assuming a random distribution. With a total of 1,268 reads covering ~33 kb of assembled sequence, the expected number of reads in each terminal region was ~134. The actual numbers of sequences were 132 and 134. Thus, the overall read density was precisely in line with what was expected but, instead of being randomly distributed across the subterminal regions, the majority of reads were derived from the termini themselves.

### Distribution of breaks near ends of hydrodynamically-sheared chromosomal DNAs

To determine if bias against subterminal breakage is responsible for the poor representation of telomeric DNA in fungal genome sequences, we downloaded raw sequence reads from several fungal genome projects with the goal of mapping shearing breakpoints relative to the chromosome ends. Unfortunately, telomeres were inadequately represented in the majority of fungal genome assemblies and, when they were present, the telomeric contigs were either too short to provide meaningful information, or they consisted of highly repetitive sequences that prevented unequivocal mapping of breaks (results not shown). The only exception was the *Neurospora crassa *assembly. Fortunately, targeted sequencing and genome finishing efforts have provided complete sequence information for several *N. crassa *chromosome ends [6]. In addition, this organism contains a modest amount of repetitive DNA, so that most sequence reads could be unambiguously assigned to unique chromosomal loci.

**Figure 5:**
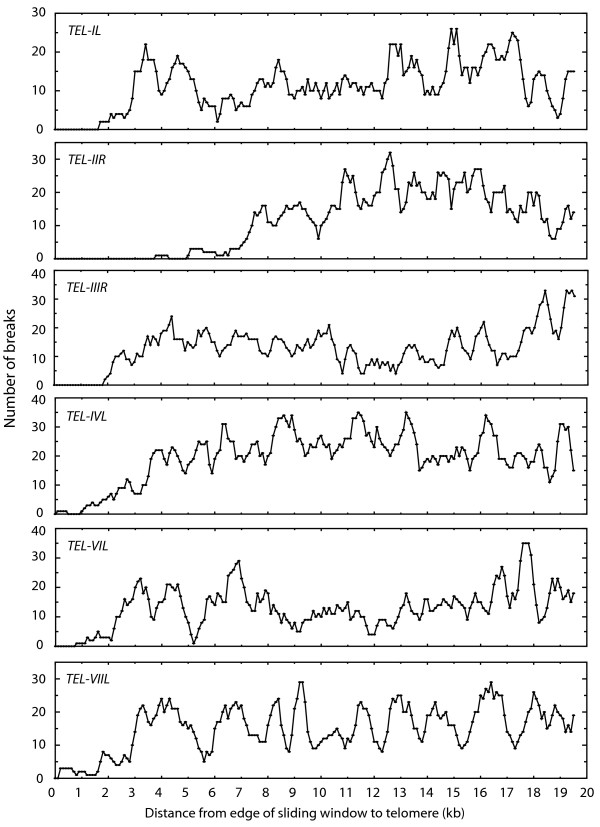
**Distribution of telomere-proximal breaks in sheared genomic DNA from *Neurospora crassa***. Trace archive data from the *N. crassa *genome sequencing project were retrieved from NCBI. BLAST searches were used to identify raw sequence reads derived from telomere-proximal regions. The positions of DNA breaks were determined by mapping the starts of the reads onto telomeric contigs that had been fully assembled through targeted finishing efforts. Shown are the results of a sliding window analysis of break distribution near to six telomeres. Window size was 500 bp and step size was 100 bp. Black line = break distribution in the circular molecular; red line = distribution in the linear molecule. The scale at the bottom represents the distance from the nearest edge of the window to the telomere.

Breaks that occurred within 20 kb of a telomere were identified by using six telomeric sequence contigs to search a BLAST database containing the raw sequence reads. Repetitive sequences were subjected to further tests to be sure that they were derived from the target region (see Materials and Methods). Breaks were then mapped using the same procedure employed for the cosmid assemblies. Four of the six chromosome ends analyzed had no breaks within 1 kb of their respective telomeres and, for three of these, the first breaks were more than 2 kb away (Table 1 and Figure 5). Even though three ends did have breaks within the terminal 2 kb, these regions still had a break density that was more than 10-fold lower than internal sequences (Table 1). As the windows were moved toward the centromeres, the density of breaks steadily increased (Figure 5). However, unlike the situation with the cosmid DNA, there were no obvious peaks in break density in the subterminal regions. Sliding window analyses were not performed on eight telomeres either because they remained unlinked to the assembly, or because they were present on contigs that were significantly shorter than 20 kb. Nevertheless, use of the corresponding eight TelContigs to search the raw reads database revealed only two overlapping sequences, again pointing to a paucity of breaks near to chromosome ends.

**Table 1:** Distribution of breaks near to *N. crassa *telomeres

Telomere	Nearest break to telomere	Breaks within 1 kb from telomere	Breaks within 2 kb from telomere	Average no. breaks in 2 kb window^A^
TEL-IL	2.1 kb	0	0	44
TEL-IIR	4.2 kb	0	0	45
TEL-IIIR	2.3 kb	0	0	50
TEL-IVL	0.5 kb	1	5	77
TEL-VIL	1.2 kb	0	4	51
TEL-VIIL	0.6 kb	3	6	60

^A. ^Measured across the entire 20 kb terminal region.

The *N. crassa *genome sequence was derived from clones with insert sizes of ~4 kb (plasmid reads) and 40 kb (fosmid reads). Shearing DNA to an average size of 40 kb is expected to produce very large shear-resistant regions. To test this prediction, we examined the distribution of breaks associated with these large insert clones. Mapping fosmid read starts revealed that shearing the DNA to an average of 40 kb produced a total of only 13 breaks within 20 kb of the six telomeres analyzed. With a total of ~30,200 sequence reads having been derived from fosmids, the expected number of breaks within 20 kb of the six telomeres (assuming a genome size of 39.9 Mb and a random distribution of breaks) is ~91. Thus, as expected, the sizes of the shearing resistant regions increased in proportion with the average length of sheared fragments.

### Overrepresentation of terminal fragments in fungal genome sequencing projects

To assess whether terminal fragments tend to be overrepresented in actual genome sequencing projects, we determined the number of clones derived from each of the 14 *N. crassa *telomeres. The average depth of shotgun sequence coverage was ~20×, so, barring the operation of counterselective forces, each telomere should be represented by approximately 20 sequences. As shown in Additional file 1, Table S1, only three telomeres were significantly overrepresented, five telomeres were represented by fewer clones than expected and six were in line with expectation. These data would appear to contradict the results obtained with Cos7-2-1. However, clones representing telomeres 13 and 14 were so abundant, that we were led to consider the possibility that telomeric fragments actually had been superabundant in the initial sheared fragment population but that most had experienced counterselection during cloning in *E. coli*. This scenario seemed plausible because the telomere-adjacent sequences were highly AT-rich - a property that leads to poor cloning efficiency in *E. coli*. In addition, at least 6× of the shotgun sequence was from clones grown in a methylation-restrictive *E. coli *host [7] which would have excluded cloning of the telomeric DNA fraction that has been shown to be methylated [8]. Therefore, to address these possible issues, we examined the representation of terminal fragments among fosmid reads. By virtue of their low copy number, fosmids often mitigate problems with the cloning of troublesome sequences. In addition, the *N. crassa *fosmid library was propagated in a methylation-persmissive *E. coli *host [7]. The total number of sequences generated from fosmid clones provided only ~0.33× coverage of the genome and, therefore, very few telomeres should have been captured in the fosmid sequences. However, as shown in Additional file 1, Table S1, nine telomeres were represented by multiple reads, with eight of them having been captured at least four times - more that 10-times the expected number. Furthermore, three telomeres were overrepresented by a factor of more than 30.

Telomeric reads were also superabundant in several other fungal genome sequencing projects. TERMINUS [1] identified complete sets of telomeres for *Aspergillus nidulans, Cochliobolus heterostrophus *and *Magnaporthe oryzae *and, in each case, the majority of telomeres were significantly overrepresented (Additional file 1, Table S1). Evidence of telomere superabundance was also seen for *Sclerotinia sclerotiorum *and the fungus-like oomycetes *Phytophthora ramorum *and *Phytophthora sojae*, although in the case of *S. sclerotiorum*, the overrepresentation was not significant for most telomeres. The *Ustilago maydis *genome project was exceptional because most telomeric fragments appeared to be systematically underrepresented. In line with what was observed for Neurospora, telomeres tended to be even more greatly overrepresented among the fosmids. The most extreme case was *Magnaporthe oryzae *where the vast majority of telomeric reads were derived from fosmid clones.

## Discussion

Here, we provide direct experimental evidence that the ends of DNA molecules are resistant to hydrodynamic shearing and we show that this creates an uneven representation of sequencing templates. As long ago as the late 1970's, Iyengar and Quave predicted that DNA termini would be resistant to shearing [4] but until now their hypothesis had remained untested. Indeed, at the time they stated that "the distribution of DNA breaks along the DNA strand is unknown, and there is no real world experiment which could directly determine this distribution" [5]. However, with the advent of high throughput DNA sequencing technology, this is now possible.

In addition to confirming a bias against subterminal breakage, we were able to quantify its impact on the assembly of DNA termini. Twelve-fold sequence coverage of a simple, linear DNA molecule that had been sheared and size-selected using conditions typical of a genome sequencing project revealed that no breaks had occurred within 1.4 kb of either DNA terminus. This distance was similar to the average length of the break-free regions at the ends of six Neurospora chromosomes (1.7 kb) and, therefore, the cosmid data appear to be fairly representative of what to expect in a fungal genome sequencing project.

As is standard procedure for such projects, short sequence contigs (in this case < 2 kb) were not included in the released Neurospora genome sequence. Therefore, in order for the telomeric sequence reads (average length: 745 bp) to be represented in the genome sequence, they must first be extended by overlapping sequences to a length of at least 2 kb. For this to happen, however, there would need to be breaks within ~650 bp of the chromosome ends. As shown here, the frequency of breakage decreased significantly near to the Neurospora chromosome ends, with only four breaks having resulted in reads that overlapped with a telomeric contig. One of these breaks resulted in sequence that allowed TEL-VIIL to be successfully assembled. However, while the others did provide sequence that overlapped with telomeric reads, a lack of additional breaks with the terminal 2 kilobases prevented further extension of these contigs which were consequently disqualified from incorporation into the assembly based on the minimum length criterion.

In contrast with our findings, Oefner and coworkers were able to generate complete assemblies of ~3.5 and 7.7 kb linear DNA fragments that had been hydrodynamically sheared [2]. However, in their study, the sizes of the fragments produced after shearing were quite different to what is used for genome sequencing (5-10% were < 400 bp) and, consequently, the sizes of their shear-resistant regions would have been quite small (likely shorter than most read lengths). In addition, they did not include a size-selection step, so small terminal fragments were not systematically excluded. Even so, inspection of subclone distribution in their assemblies still revealed a complete absence of breaks within ~1 kb of the fragment ends and showed that the termini were only assembled because the clone libraries contained rare fragments that allowed the "end sequences" to be linked to the major internal contigs [2].

A second prediction from the computer models of Iyengar and Quave was that shearing would result in an exceptionally large number of breaks at a distance from each end that corresponds to the average fragment size [4]. This prediction was also borne out by our cosmid data which showed clear peaks in break density near each end of the linear molecule (Figure 3A). Similar peaks were absent in the *N. crassa *telomere regions but this was probably because the genome sequence was acquired from DNA that had been sheared to three different size ranges (~4 kb, ~10 kb and ~40 kb).

An interesting and unexpected consequence of the subterminal breakage hotspots, was a dramatic overrepresentation of sequences derived from the ends of DNA molecules. This discovery was especially intriguing because we had previously observed that certain telomeres tended to be highly enriched among the raw reads from several fungal genome sequencing projects [1,6,9]. At the time, we were surprised by this observation because telomeric DNA needs to be end-repaired prior to cloning whereas most sheared ends do not [10] and, therefore, we expected telomeres to be less efficiently cloned than internal DNA. However, we show here that telomere superabundance can be fully explained by the uneven distribution of shearing points - in essence, additional terminal fragments are produced in compensation for the missing subterminal subclones. This principle predicts that the proportional representation of terminal clones will increase as the lengths of the shear resistant regions increase. This prediction was borne out by the fosmid data.

It is worth pointing out that Oefner et al. also reported an overabundance of terminal sequences after shearing and assembling two different linear restriction fragments [2]. They attributed this to preferential cloning of enzymatically-cleaved DNA termini. However, based on the evidence of shearing bias in their experimental data, it seems more likely that this, too, resulted from refractoriness to subterminal breakage.

Our cosmid data also provided support for Iyengar and Quave's prediction that the frequency of breaks near the termini would oscillate with a pattern resembling Gibb's phenomenon, which describes how Fourier sums alternately overshoot and undershoot a discontinuous function at jump discontinuities [11,12]. The break density at positions corresponding to the average fragment size clearly overshot the average number of breaks measured across the entire cosmid insert and these overshoots were followed by smaller, but clearly discernable, undershoots. The physical basis for this behavior is easily explained by the presence of breakage hotspots because these will produce a high density of "secondary" ends in a narrow sequence window. Accordingly, one would expect the regions adjacent to these secondary ends also to be resistant to breakage. With Gibb's phenomenon, the amplitude of the oscillations decreases as the distance from the discontinuity increases, although they never disappear. We were unable to detect further oscillations in the cosmid data due to the inherent fluctuations in break density that comes with a relatively low level of sequence coverage.

## Conclusions

Based on our findings, we conclude that a systematic bias against subterminal breakage during DNA shearing is a major reason for the lack of telomere sequences in fungal genome assemblies. Specifically, the paucity of breaks at appropriate distances from the chromosome ends prevents the extension of telomeric sequence contigs, which are then excluded from the genome assembly due to their short length. Although there may be additional barriers to the incorporation of fungal telomeres such as the existence of subtelomeric sequences duplicated at multiple chromosome ends, as well as other types of dispersed repeats [9,13,14], analyses of telomere regions from several fungi suggest that these usually are minor issues. In addition to assembly problems, there were some fungal genome projects for which TERMINUS identified very few telomeres among the raw sequence reads. Possible reasons for this include an inefficient end-repair step or counterselection of telomeric clones during cloning in *E. coli*.

Shearing bias is likely to affect telomere assembly for any organism with short telomere tracts but is not expected to be a problem in vertebrates, insects and plants, whose telomeres can be more than 10 kb in length [15-17]. In these cases, the repeat tract will extend beyond the shearing resistant regions so, although complete reconstruction of the telomere tracts may be prevented, the proximal portion of each telomere should be represented in the final assembly. Any failure to incorporate telomeres in the genome sequences for these organisms is, therefore, most likely due to other factors.

In the context of a whole genome assembly, the total size of subterminal gaps resulting from break deficiency is extremely small. However, if the telomeres cannot somehow be incorporated, then it is not be possible to determine which of the internal contigs are located near to the chromosome ends - at least without additional experimentation. This potentially could lead to some very interesting biology being overlooked because the terminal regions of fungal chromosomes tend to be regions of enhanced genomic variation [18-20] and, in several fungi, they harbor genes that are predicted to confer adaptive advantages [9,14,18,21]. Therefore, should it be desirable to incorporate telomeres into genome assemblies, this would best be accomplished by using the TERMINUS program [1] to identify sequence templates that span key gaps between the telomeres and the genome assembly and then acquiring the missing sequences by primer walking using the corresponding plasmid or fosmid templates.

## Methods

### Preparation and shearing of circular and linearized forms of cosmid DNA

Cosmid 7-2-1 was prepared using a standard alkaline lysis procedure. An aliquot of DNA was treated with *Swa*I restriction enzyme which cuts once within the clone, creating blunt ends. Restriction enzyme buffer (but no enzyme) was added to the remaining DNA sample to ensure that the salt concentrations in the circular and linear DNA samples were equivalent. After digestion, the DNA samples were filtered through 0.22 μm Spin-X columns (Costar, Corning, NY) to remove any particulate matter. Shearing was performed using a Hydroshear^® ^machine (GeneMachines^®^, San Carlos, CA) with a standard shearing assembly, using speed settings of 12 for the "circular" DNA sample and one aliquot of linearized DNA and 6 for another aliquot. Following shearing, the DNA samples were precipitated and resuspended in 1× bromophenol blue loading dye solution in TE. The samples were size-fractioned by electrophoresis through 0.7% agarose in 0.5× TBE, stained with ethidium bromide and viewed under long wavelength UV light. For the circular and linear samples sheared at speed setting 12, fragments of between 3.8 and 4.2 kb were excised from the gel; for the sample sheared at the higher speed (setting 6), fragments of 1.8 to 2.2 kb were collected. DNA was recovered from the gel slices using the Qiaquick kit (Qiagen, Valencia, CA) and eluted in 40 μl elution buffer. Approximately, 200 ng of each sample was mixed with ~50 ng of phosphatase-treated pBC KS^+ ^(Stratagene, La Jolla, CA) that had been linearized by treatment with *Eco*RV and *Sma*I. Ligation was performed overnight at 12°C in a total reaction volume of 5 μl, in the buffer supplied by the manufacturer (New England Biolabs, Ipswich, MA). One microliter of ligation mix was transformed into T1-resistant DH5α cells (Invitrogen, Carlsbad, CA) which were then spread on LB agar + 12.5 μg/ml chloramphenicol.

Transformants were picked to LB+Hogness freezing medium+12.5 μg/ml chloramphenicol in 384-well microtiter plates. For DNA preparation, frozen stocks were picked to 1.2 ml of YT broth + chloramphenicol in deep 96-well plates and cultured with shaking for 20 h at 37°C. DNA was extracted by alkaline lysis, cell debris was removed by filtration through 96-well filter plates (Whatman, Florham Park, NJ). The DNA was precipitated by adding 0.7 vol. of isopropanol, the pellets were washed with 70% EtOH and re-dissolved in 40 μl T0.1XE (Tris-HCl, pH8.0; 0.1 mM EDTA). Two microliters of each sample was used for sequencing.

#### DNA sequencing

Dye terminator sequencing was performed using DTCS chemistry (Beckman Coulter, Fullerton, CA) and the sequence data were obtained on Beckman CEQ2000 and CEQ2000XL sequencing machines. Sequence quality was assessed using Phred [22,23] and reads with fewer than 100 high quality bases (≥ Phred20) were discarded. The remaining high quality reads were assembled using Phrap http://www.phrap.org.

#### Determination of insert size distribution

DNA fragments in the range 3.8 to 4.2 kb were selected for cloning. However, there was a possibility that cloning is biased toward recovery of smaller fragments. To gain a better estimate of insert size distribution, we mapped the start points of sequences from each end of plasmid against the assembled cosmid sequence. In brief, a Perl script was used to identify the cloning site and capture the first 24 nucleotides of the insert. These sequences were used to search the assembled cosmid sequence. To minimize errors caused by chimeric clones, results were checked to ensure that the two ends of a clone matched in opposite orientation. Inserts possessing a size more than twice the average were also considered chimeras and excluded from the analysis.

#### Sliding window analysis of break distribution on cosmid templates

Break distributions were examined by counting breaks in a 1 kb window that was slid in 100 bp increments across a 20 kb region spanning the *Swa*I cut site. For the circular cosmid, the starting position was with the trailing edge of the window 10 kb away from the *Swa*I site on one side. The window was then slid toward and across the *Swa*I site, with the counts being stopped when the leading of the window reached a position 20 kb on the other side. Two windows were used for the linearized template. The starting position for each one was with the trailing edge at the *Swa*I site. The windows were then slid away in opposite directions until their leading edges reached the respective 10 kb marks. Breaks within the cosmid backbone were included in this analysis.

#### Mapping breakpoints in the sheared DNA used in the Neurospora crassa genome sequencing project

Raw sequence reads and ancillary files were retrieved from the NCBI Trace Archive http://www.ncbi.nlm.nih.gov/Traces/trace.cgi?. To be sure that we analyzed sequences derived from sheared DNA, only reads obtained in the whole genome shotgun (WGS) phase of the whole genome project were processed. These reads were first used as queries in BLAST searches of a database containing 20 kb of sequence from each of six chromosome ends. Sequences yielding positive matches were then used to search the whole genome assembly to confirm that they were single-copy. Repetitive sequences were further processed to try and obtain corroboratory evidence that they were truly derived from the region under study. The criteria used for acceptance of such reads required that they were ≥ 98% identical to the region of interest, or the corresponding mate-pair reads were unique sequences that could be unambiguously assigned to the region in question. Once all of the reads that matched a given telomere had been identified, the positions of the original DNA breaks were ascertained by examining the starting coordinate of the BLAST match and mapping this back to fully assembled telomere sequence that had been derived through finishing efforts. The distributions of breaks relative to the telomere were then determined using a sliding window procedure in which a 500 bp window was slid in 100 bp increments across the telomere region and the number of breaks occurring within the window was totaled for each position.

## Authors' contributions

SLS prepared, sheared, subcloned and sequenced the cosmid DNAs. MLF assembled the sequence data and performed the bioinformatics analyses and wrote the manuscript. Both SLS and MLF have read the final manuscript and approve it for publication.

## Supplementary Material

Additional File 1 **Table S1. Telomere representation among shotgun sequencing reads**. The table lists the numbers of telomeric reads derived from plasmid and fosmid clones in several fungal genome sequencing projects. Click here for file
