# Supplementary material for: Systematic overrepresentation of DNA termini and underrepresentation of subterminal regions among sequencing templates prepared from hydrodynamically sheared linear DNA molecules
Source: BMC Genomics. 2010 Feb 2;11:87. doi: 10.1186/1471-2164-11-87 (PMC2824731; doi:10.1186/1471-2164-11-87)
Supplement: Additional File 1 — Table S1. Telomere representation among shotgun sequencing reads. The table lists the numbers of telomeric reads derived from plasmid and fosmid clones in several fungal genome sequencing projects. [file 1471-2164-11-87-S1.DOC]

**Supplemental Table 1.** Telomere representation among shotgun sequencing reads.

| **Fungus** | **Coverage** | **TelContig^A^** | **Total reads** | **Fosmid^B^ reads** |
| --- | --- | --- | --- | --- |
| ***Neurospora crassa*** | **20X (P+F)**^C^ | 1 | 2 | 0 |
| **Telomeres = 14** | **0.33X (F)** | 2 | 3 | 0 |
|  |  | 3 | 3 | 0 |
|  |  | 4 | 6 | 0 |
|  |  | 5 | 9 | 9 |
|  |  | 6 | 21 | 7 |
|  |  | 7 | 22 | 12 |
|  |  | 8 | 23 | 2 |
|  |  | 9 | 26 | 9 |
|  |  | 10 | 27 | 14 |
|  |  | 11 | 28 | 4 |
|  |  | 12 | 36*^D^ | 7 |
|  |  | 13 | 47* | 23 |
|  |  | 14 | 48* | 0 |
| ***Aspergillus nidulans*** | **10X (P+F)** | 1 | 38* | 25 |
| **Telomeres = 16** | **0.22X (F)** | 2 | 40* | 17 |
|  |  | 3 | 43* | 17 |
|  |  | 4 | 44* | 20 |
|  |  | 5 | 45* | 26 |
|  |  | 6 | 48* | 24 |
|  |  | 7 | 50* | 25 |
|  |  | 8 | 54* | 20 |
|  |  | 9 | 60* | 18 |
|  |  | 10 | 70* | 24 |
|  |  | 11 | 10 | 10 |
|  |  | 12 | 11 | 10 |
|  |  | 13 | 8 | 4 |
|  |  | 14 | 12 | 8 |
|  |  | 15 | 29* | 7 |
|  |  | 16 | 70* | 24 |
| ***Magnaporthe oryzae*** | **7X (P+F)** | 1 | 13 | 13 |
| **Telomeres = 14** | **0.1X (F)** | 2 | 17* | 9 |
|  |  | 3 | 31* | 30 |
|  |  | 4 | 32* | 32 |
|  |  | 5 | 34* | 25 |
|  |  | 6 | 36* | 35 |
|  |  | 7 | 45* | 44 |
|  |  | 8 | 46* | 45 |
|  |  | 9 | 48* | 47 |
|  |  | 10 | 83* | 77 |
|  |  | 11 | 44* | 39 |
|  |  | 12 | 15 | 12 |
|  |  | 13 | 29* | 25 |
|  |  | 14 | 6 | 4 |
| ***Sclerotinia sclerotiorum*** | **10X (P+F)** | 1 | 3 | 0 |
| **Telomeres = 16** |  | 2 | 5 | 2 |
|  |  | 3 | 5 | 3 |
|  |  | 4 | 5 | 1 |
|  |  | 5 | 6 | 0 |
|  |  | 6 | 6 | 2 |
|  |  | 7 | 7 | 1 |
|  |  | 8 | 6 | 4 |
|  |  | 9 | 8 | 0 |
|  |  | 10 | 11 | 7 |
|  |  | 11 | 11 | 1 |
|  |  | 12 | 12 | 3 |
|  |  | 13 | 12 | 3 |
|  |  | 14 | 14 | 3 |
|  |  | 15 | 14 | 2 |
|  |  | 16 | 15 | 5 |
|  |  | 17 | 15 | 0 |
|  |  | 18 | 15 | 1 |
|  |  | 19 | 15 | 4 |
|  |  | 20 | 15 | 6 |
|  |  | 21 | 14 | 1 |
|  |  | 22 | 17 | 4 |
|  |  | 23 | 18 | 0 |
|  |  | 24 | 20 | 2 |
|  |  | 25 | 20 | 3 |
|  |  | 26 | 22* | 1 |
|  |  | 27 | 24* | 6 |
|  |  | 28 | 22* | 5 |
|  |  | 29 | 24* | 3 |
|  |  | 30 | 23* | 3 |
|  |  | 31 | 2 | 0 |
| ***Ustilago maydis*** | **10X (P+F)** | 1 | 2 | 0 |
| **Telomeres = 46** | **1.2X (F)** | 2 | 2 | 1 |
|  |  | 3 | 2 | 0 |
|  |  | 4 | 2 | 0 |
|  |  | 5 | 2 | 0 |
|  |  | 6 | 2 | 1 |
|  |  | 7 | 2 | 2 |
|  |  | 8 | 2 | 1 |
|  |  | 9 | 3 | 3 |
|  |  | 10 | 3 | 3 |
|  |  | 11 | 4 | 2 |
|  |  | 12 | 5 | 1 |
|  |  | 13 | 5 | 2 |
|  |  | 14 | 5 | 1 |
|  |  | 15 | 6 | 6 |
|  |  | 16 | 7 | 4 |
|  |  | 17 | 8 | 5 |
|  |  | 18 | 9 | 6 |
|  |  | 19 | 10 | 6 |
|  |  | 20 | 10 | 6 |
|  |  | 21 | 12 | 4 |
|  |  | 22 | 12 | 4 |
|  |  | 23 | 16 | 11 |
|  |  | 24 | 18 | 7 |
|  |  | 25 | 2 | 1 |
|  |  | 26 | 6 | 4 |
|  |  | 27 | 2 | 2 |
|  |  | 28 | 3 | 1 |
|  |  | 29 | 2 | 1 |
|  |  | 30 | 4 | 2 |
| ***Cochliobolus heterostrophus*** | **10X (P+F)** | 1 | 2 | - |
| **Telomeres = 15** |  | 2 | 2 | - |
|  |  | 3 | 3 | - |
|  |  | 4 | 3 | - |
|  |  | 5 | 9 | - |
|  |  | 6 | 10 | - |
|  |  | 7 | 12 | - |
|  |  | 8 | 15 | - |
|  |  | 9 | 16 | - |
|  |  | 10 | 18 | - |
|  |  | 11 | 20 | - |
|  |  | 12 | 20 | - |
|  |  | 13 | 21 | - |
|  |  | 14 | 21* | - |
|  |  | 15 | 25* | - |
|  |  | 16 | 27* | - |
|  |  | 17 | 27* | - |
|  |  | 18 | 27* | - |
|  |  | 19 | 28* | - |
|  |  | 20 | 29* | - |
|  |  | 21 | 30* | - |
|  |  | 22 | 33* | - |
|  |  | 23 | 35* | - |
|  |  | 24 | 36* | - |
|  |  | 25 | 40* | - |
|  |  | 26 | 43* | - |
|  |  | 27 | 44* | - |
|  |  | 28 | 45* | - |
|  |  | 29 | 115* | - |
|  |  | 30 | 185* | - |
| ***P. ramorum*** | **7.7X (P+F)** | 1 | 3 | - |
| **Telomeres = 11-14 (estimated)** |  | 2 | 6 | - |
|  |  | 3 | 6 | - |
|  |  | 4 | 7 | - |
|  |  | 5 | 8 | - |
|  |  | 6 | 8 | - |
|  |  | 7 | 11 | - |
|  |  | 8 | 13 | - |
|  |  | 9 | 14 | - |
|  |  | 10 | 15 | - |
|  |  | 11 | 19* | - |
|  |  | 12 | 19* | - |
|  |  | 13 | 24* | - |
|  |  | 14 | 23* | - |
|  |  | 15 | 25* | - |
|  |  | 16 | 26* | - |
|  |  | 17 | 27* | - |
|  |  | 18 | 32* | - |
|  |  | 19 | 36* | - |
|  |  | 20 | 39* | - |
|  |  | 21 | 18* | - |
|  |  | 22 | 10 | - |
|  |  | 23 | 5 | - |
|  |  | 24 | 2 | - |
|  |  | 25 | 2 | - |
|  |  | 26 | 39* | - |
|  |  | 27 | 2 | - |
|  |  | 28 | 140* | - |
| ***P. sojae*** | **9X (P+F)** | 1 | 2 | - |
| **Telomeres = 11-14 (estimated)** |  | 2 | 3 | - |
|  |  | 3 | 4 | - |
|  |  | 4 | 14 | - |
|  |  | 5 | 24* | - |
|  |  | 6 | 24* | - |
|  |  | 7 | 28* | - |
|  |  | 8 | 28* | - |
|  |  | 9 | 29* | - |
|  |  | 10 | 29* | - |
|  |  | 11 | 33* | - |
|  |  | 12 | 40* | - |
|  |  | 13 | 42* | - |
|  |  | 14 | 48* | - |
|  |  | 15 | 46* | - |
|  |  | 16 | 51* | - |
|  |  | 17 | 58* | - |
|  |  | 18 | 62* | - |
|  |  | 19 | 58* | - |
|  |  | 20 | 6 | - |
|  |  | 21 | 67* | - |
|  |  | 22 | 67* | - |
|  |  | 23 | 72* | - |
|  |  | 24 | 86* | - |
|  |  | 25 | 88* | - |
|  |  | 26 | 117* | - |

^A^. TelContig identified by TERMINUS.

^B^. ‘-‘ = data not available.

^C^. P = plasmid reads; F = fosmids reads.

^D^. Indicates that telomeric reads were significantly over-represented based on the Chi‑square goodness of fit test (P=0.05).
